# Association of Metabolic Dysfunction-Associated Fatty Liver Disease and Liver Stiffness With Bone Mineral Density in American Adults

**DOI:** 10.3389/fendo.2022.891382

**Published:** 2022-06-30

**Authors:** Hejun Li, Hengcong Luo, Ying Zhang, Lisi Liu, Rong Lin

**Affiliations:** ^1^ Department of Endocrinology, The Third Affiliated Hospital of Guangzhou Medical University, Guangzhou, China; ^2^ Department of Nephrology, The Third Affiliated Hospital of Guangzhou Medical University, Guangzhou, China

**Keywords:** Metabolic dysfunction-associated fatty liver disease, Osteoporosis, bone mineral density, National Health and Nutrition Examination Survey, cross-sectional study

## Abstract

**Contest:**

The relationship between metabolic dysfunction-associated fatty liver disease (MAFLD) and liver stiffness and bone mineral density (BMD) remains unclear.

**Objectives:**

We aimed to investigate the association between MAFLD and liver stiffness and BMD in the United States population.

**Methods:**

A cross-sectional study among 2031 participants over 50 years old in the National Health and Nutrition Examination Survey (NHANES) 2017-2018 was performed. All patients underwent vibration controlled transient elastography (VCTE) and dual-energy x-ray absorptiometry (DXA). The linear and logistic regression model were used to analyze the association between the MAFLD and liver stiffness and osteoporosis, with adjustments for known covariates. Furthermore, the sensitive analyses were conducted to explore the relationship between MAFLD and liver stiffness and whole osteoporosis (include femoral and lumbar osteoporosis).

**Results:**

MAFLD was prevalent in the study population, with a prevalence of 50.9% for men and 40.7% for women. The multiple linear models demonstrated positive associations between MAFLD and liver stiffness and total femur BMD, femur neck BMD, trochanter BMD, intertrochanter BMD. In multiple logistic regression models, both MAFLD and significant liver fibrosis were negatively associated with femoral osteoporosis (OR=0.41, 95% CI: 0.27 to 0.63; OR=0.67, 95% CI: 0.33-1.37, respectively). Nonetheless, when BMI was adjusted, the association between MAFLD and liver stiffness and osteoporosis became insignificant. Besides, as showed in the sensitive analyses, the relationship between MAFLD and liver stiffness and whole osteoporosis were stable.

**Conclusions:**

These results suggest that MAFLD and liver stiffness were associated with higher femoral and lumbar bone mineral density in individuals aged over 50 years. But the results may be confounded by BMI.

## Introduction

Non-alcoholic fatty liver disease (NAFLD) is the most prevalent chronic liver disease worldwide, affecting approximately 25% of general population worldwide (1). The incidence of NAFLD is closely related to obesity (2), but may also affect people with normal weight (3). In the United States, the annual medical costs directly attributable to NAFLD are estimated at more than $100 billion (4). Since closely associated with metabolic diseases, NAFLD is also an independent risk factor for type 2 diabetes mellitus (T2DM), cardiovascular disease and mortality (5), and as the incidence of metabolic diseases increases, the diagnosis of NAFLD may become obsolete. Therefore, an international panel of experts recently proposed a consensus to define the fatty liver as metabolic dysfunction-associated fatty liver disease (MAFLD) (6, 7). The new criteria no longer exclude those with alcoholic fatty liver disease or viral hepatitis, but place greater emphasis on metabolic factors in the disease (6, 7). Although, the prevalence of MAFLD and the risk of advanced fibrosis were parallel with NAFLD (8), a recent real-world study based on NHANES III by Lin et al (9) confirmed that patients with MAFLD had more metabolic disorders than those with NAFLD. It may suggest that the new criteria may able to identify individuals with metabolic disorders. Osteoporosis, the most common chronic metabolic bone disease, is characterized by reduced bone density, deterioration of microstructure, and increased bone fragility (10). In the United States, more than 15 percent of men and a quarter of women over the age of 50 are diagnosed with osteoporosis and it causes 1.5 million fractures each year (11, 12). Increasing studies have characterized bone as an endocrine organ (13). Osteoporosis in the elderly is considered to be a disorder of bone metabolism. In addition to advanced age, osteoporosis is also affected by many factors, such as smoking, weight loss, lack of exercise, ect (14–17). MAFLD is a multi-system disease that includes overweight, T2DM, hypertension, and several metabolic disorders situation, which can affect multiple body parts. Therefore, it may exert detrimental effects on some extra-hepatic organs, such as the kidneys, cardiovascular system (18, 19). Previous studies have suggested that hyperlipidemia may increase the risk of osteoporosis (20). However, components of the MAFLD, such as obesity, insulin resistance (IR) or body mass index (BMI) (21, 22), are considered as protective factors for osteoporosis. Therefore, the association between MAFLD and liver fibrosis and BMD is still unclear. To our knowledge, little is known the association between MAFLD and liver fibrosis and BMD. Therefore, we used data from NHANES, a general population representative of men and women in the United States, to conduct a cross-sectional study to analyze the possible association between MAFLD and liver fibrosis and BMD.

## Materials and Methods

### Study Participants

**Figure 1:**
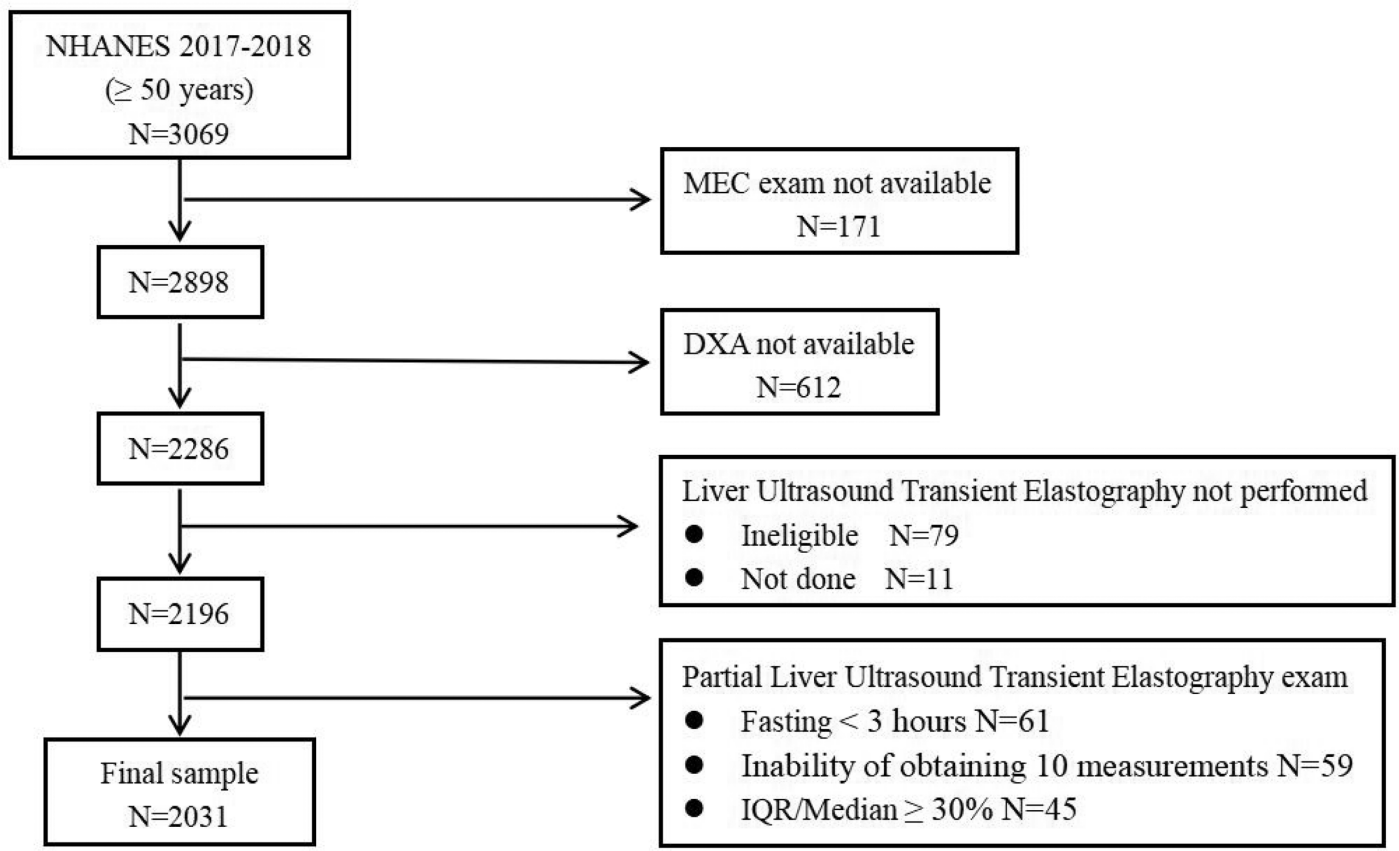
Flow diagram of the research study design.

Data from NHANES 2017-2018 were used for this study. NHANES is an annual cross-sectional survey conducted by the National Center for Health Statistics (NCHS) of the Centers for Disease Control and Prevention (CDC) (23). The data were collected using a complex multi-stage probabilistic sampling design and a complex weighting scheme that over-sampled certain ethnic and age groups and ultimately provided a representative sample of the non-institutionalized civilian population of the United States. 3,069 adults (over 50 years) participated in the NHANES 2017-2018 survey, and we excluded those who did not have laboratory testing at a mobile examination center (N=171). In addition, we also excluded those with missing data on Dual-Energy X-ray Absorptiometry (DXA) (N=612) or Vibration controlled transient elastography (VCTE) N=255). Therefore, the final study sample enrolled 2031 adults with complete data (**Figure 1**). Because all participants had signed written informed consent and their personal information was fully de-identified, our study was granted an exemption from the Institutional Review Board.

### Clinical and Laboratory Data

We collected demographic, anthropometric, related laboratory, and self-reported questionnaire data. Demographic variables, including age, gender (Male, Female), and race/ethnicity (Mexican American, Other Hispanic, Non-Hispanic White, Non-Hispanic Black, and Other Race), educational level (Below high school, High school and above), marital status (Married/Living with partner, Widowed/Divorced/Separated/Never married) were ascertained by questionnaire. Anthropometric indicators include body mass index (BMI) and waist circumference (WC). The indexes measured included glycated hemoglobin A1c (HbA1c), glucose, insulin, triglyceride, total cholesterol, high-density lipoprotein cholesterol (HDL-C), alanine aminotransferase (ALT), aspartate aminotransferase (AST), alkaline phosphatase (ALP), gamma glutamyl transferase (GGT), total bilirubin, platelet count, creatinine, total serum calcium, serum phosphorus, albumin, uric acid, high sensitivity c-reactive protein (HS-CRP), as previously described (24). Blood pressure is measured by a blood pressure inspector who has passed an accredited training program. Participants were measured three times after sitting for 5 minutes and to determine the maximum dilation level (MIL). We took the average of the three measurements as the representative blood pressure of the participant. Hypertension was defined by the presence of one of two criteria (25): systolic blood pressure ≥140 mmHg and/or diastolic blood pressure ≥90 mmHg or currently taking antihypertensive medications. Diabetes mellitus was defined by the presence of one of the following conditions: a) self-reported diabetes; b) fasting blood glucose ≥126mg/dL; c) HBA1c level ≥6.5%; d) use of anti-diabetic drug, including insulin (26). Homoeostasis model assessment of insulin resistance (HOMA-IR) was conducted to estimate insulin resistance using the equation by Matthews et al. (27).

We categorized drinking status as drinker or never drinker, according to the following questions “Ever had a drink of any kind of alcohol?”. Besides, we categorized smoking status as smokers or never smokers, according to the following questions “Smoked at least 100 cigarettes in life?”.

### Vibration Controlled Transient Elastography

Although liver biopsy is recommended as the gold standard for diagnosing fatty liver disease, it may not be appropriate in large population studies due to its invasive nature. VCTE is a noninvasive, convenient, and widely available technique that uses controlled attenuation parameter (CAP) and liver stiffness measurement (LSM) to define fatty liver and fibrosis (28). In the NHANES 2017-2018 cycle, health technicians completed a 2-day training program with survey staff and an expert FibroScan^®^ Technician (reference examiner). Training will be provided by NCHS personnel, Westat, and an Echosens expert trainer. The training included an overview of the component, demonstrations conducted by the reference examiner with volunteer subjects. The reference examiner reviewed and demonstrated the proper technique of the FibroScan^®^ examination. The elastography measurements were obtained in the NHANES Mobile Examination Center (MEC), using the FibroScan^®^ model 502 V2 Touch equipped with a medium (M) or extra-large (XL) wand (probe). Supervised practice exercises followed, conducted with several volunteer subjects. The reference examiner would certify the health technician after observing 3 satisfactory exams. The inter-rater reliability (health technician compared with reference examiners, n=32) was 0.86 for stiffness, and 0.94 for CAP-steatosis. CAP ≥274 dB/m indicated liver steatosis (S ≥1), as this cut-off point has 90% sensitivity in identifying any degree of hepatic steatosis (29). The cut-off for liver fibrosis (LF) were obtained from previous study (29). Significant LF, severe LF and cirrhosis were defined as LSM ≥ 8.2 KPa (fibrosis grade ≥ F2), LSM ≥ 9.7 KPa (fibrosis grade ≥ F3) and LSM ≥ 13.6 KPa (fibrosis grade ≥ F4), respectively.

### The Diagnostic Criteria for MAFLD

According to the 2020 international expert consensus statement (7), a positive diagnosis of MAFLD is based on VCTE evidence of fat accumulation in the liver (hepatic steatosis) in addition to one of the following three criteria, namely overweight/obesity, presence of T2DM mellitus (T2DM), or evidence of metabolic dysregulation. Metabolic dysregulation exhibits the following characteristics: 1) waist circumference ≥102/88 cm in Caucasian men and women; 2) blood pressure ≥130/85 mmHg or specific drug treatment; 3) plasma triglycerides ≥150 mg/dl or specific drug treatment; 4) plasma HDL-cholesterol <40 mg/dl for men and <50 mg/dl for women or specific drug treatment; 5) prediabetes (fasting glucose levels 100 to 125 mg/dl, or HbA1c 5.7% to 6.4%; 6) HOMA-IR score ≥2.5; 7) plasma high-sensitivity C-reactive protein level >2 mg/L.

### Bone Mineral Density Measurement

BMD was measured in the left hip bone using a Dual-Energy x-ray absorptiometry (DXA) scan with a fan-beam densitometer (QDR4500A; HOGICF). Pregnant women and the participants less than 50 years old or with self-reported history of radiographic contrast material in the past week or weighted over 450 pounds were ineligible for the DXA scan. Besides, participants with hip fracture, replacements or pins in both hips were also excluded. Further details of the DXA examination protocol were documented in the Body Composition Procedures Manual located on the NHANES website. According to the guidelines of the World Health Organization (WHO), the diagnostic criteria of osteoporosis or osteopenia is based on T-score results. T-scores were calculated as (BMD measured − mean BMD reference)/SD reference. Osteoporosis was defined as a T-score of BMD ≤ − 2.5, and osteopenia was defined as − 2.5 < T-score ≤ − 1. We used non-Hispanic white women between the ages of 20-29 in NHANES III as a reference group for calculating femoral BMD scores (30). The lumbar BMD T -score references for calculating were extracted from previous study (31). A T-score ≤ − 2.5 in any part of the femur, femoral neck, trochanter, intertrochanter and lumbar was diagnosed as osteoporosis.

### Statistical Analysis

Considering the complex sample design adopted by NHANES, we utilized weights in the statistical analysis to ensure that the data were representative of the entire United States non-institutionalized population. The weights were applied in accordance with the guidelines for using raw data from NHANES provided by the NCHS. Continuous variable was presented as weighted mean ± SE and classified variable as weighted percentage. The Chi-square test was performed for categorical variables and Kruskal-Wallis test for continuous variables to describe the clinical characteristics of patients with or without MAFLD. Weighted multiple linear regression analyses were conducted to analyze the association between MAFLD and BMD after adjusting for known or selected confounders. Confounders were selected for their significant association with the BMD, or a changed in effect estimate of more than 10%. To avoid multicollinearity, variance inflation factors were assessed before adjustment. The subgroup analyses were stratified by sex (male and female), age (<60, 60 to 70, and ≥70 years), abdominal obesity (yes or no), hyper-hs CRP (yes or no) and Low HDL (yes or no).

Given that the influence of fatty liver or liver fibrosis on femoral or lumbar BMD may be inconsistent. Hence, in sensitivity analysis, we excluded those without lumbar bone mineral density data and explored the relationship between MAFLD and liver fibrosis and whole osteoporosis. All analyses were performed in R software (version 3.6.1). All tests were two-tailed and P values <0.05 were considered statistically significant.

## Results

### Study Participants and Baseline Characteristics

A total of 2031 participants (1070 males and 961 females) were included in the study. **Table 1** summarizes the baseline characteristics of participants with and without MAFLD. Based on diagnostic criteria for MAFLD, 446 men and 400 women were diagnosed, with a weighted prevalence of 50.9% (95% CI, 45.3%-56.3%) and 40.7% (95% CI, 36.4%-45.1%), respectively. Compared with participants without MAFLD, those with MAFLD were more likely to be man, IR, hypertensive, diabetic, pre-diabetic, overweight and abdominal obesity. In addition, these MAFLD participants were noted to have a higher body mass index, alanine aminotransferase, alkaline phosphatase, gamma glutamyl transferase, fasting plasma glucose, fasting insulin, uric acid, HbA1c, hs-CRP, triglyceride, larger waist circumference, waist circumference and lower total cholesterol, HDL-C. As we predicted, participants diagnosed with MAFLD had a lower weighted prevalence of osteoporosis than those without MAFLD, although this outcome may be affected by BMI. A lower rate of osteoporosis observed in women with MAFLD did not reach significance.

**Table 1:** Baseline characteristics of subjects by presence of the MAFLD according to sex (N=2031).

Characteristic	Total	MAFLD (+)	MAFLD (-)	P value
Age (years)	63.08 ± 0.42	62.89 ± 0.47	63.24 ± 0.47	0.4348
Women (%)	51.15+1.11	45.59 ± 2.39	55.82 ± 1.66	0.0094**
High school and Above (%)	60.86+1.91	59.32 ± 2.29	62.15 ± 3.69	0.5816
Married or Living with partner (%)	66.66+2.11	70.94 ± 2.87	63.06 ± 2.87	0.0585
Non-Hispanic White (%)	9.43 ± 1.41	8.44 ± 1.45	10.25 ± 1.64	0.1918
Drinking habit (%)	91.99+1.21	90.54 ± 2.34	93.22 ± 1.08	0.2935
Smoking habit (%)	43.75+1.66	43.39 ± 2.19	44.05 ± 2.67	0.8593
BMI (kg/m2)	29.05 ± 0.29	32.64 ± 0.42	26.03 ± 0.25	<0.0001***
Hip circumference (cm)	101.35 ± 0.79	110.58 ± 1.04	93.62 ± 0.68	<0.0001***
Waist circumference (cm)	106.14 ± 0.45	112.69 ± 0.8	100.63 ± 0.47	<0.0001***
ALP (IU/L)	80.76 ± 0.79	82.61 ± 1.08	79.17 ± 1.11	0.0417*
Albumin (g/dL)	4.06 ± 0.02	4.06 ± 0.02	4.06 ± 0.02	0.9611
ALT (IU/L)	22.65 ± 0.57	24.89 ± 0.56	20.73 ± 1	0.0034**
GGT (IU/L)	31.35 ± 0.99	33.59 ± 1.18	29.44 ± 1.31	0.0192*
AST (IU/L)	22.38 ± 0.51	22.5 ± 0.54	22.28 ± 0.81	0.8189
Creatinine (mg/dL)	0.91 ± 0.01	0.91 ± 0.01	0.91 ± 0.01	0.9914
Phosphorus (mg/dL)	3.58 ± 0.02	3.54 ± 0.03	3.61 ± 0.02	0.0958
Total bilirubin (mg/dL)	0.49 ± 0.01	0.49 ± 0.02	0.49 ± 0.02	0.9156
Total calcium (mg/dL)	9.34 ± 0.02	9.35 ± 0.03	9.32 ± 0.02	0.3345
Total cholesterol (mg/dL)	196.67 ± 2.12	190.92 ± 3.34	201.61 ± 2.3	0.0124*
Triglyceride (mg/dL)	151.6 ± 4.26	183.41 ± 8.77	124.28 ± 3.95	<0.0001***
Uric acid (mg/dL)	5.46 ± 0.07	5.76 ± 0.07	5.21 ± 0.08	<0.0001***
Platelet count (10^9/L)	232.18 ± 3.29	233.51 ± 3.65	231.06 ± 4.27	0.6018
Osteoporosis (%)	7.3 ± 0.61	4.32 ± 0.59	9.81 ± 1.03	0.0001***
Total femur BMD (gm/cm2)	0.92 ± 0.00	0.97 ± 0.01	0.87 ± 0.01	0.0001***
Femur neck BMD (gm/cm2)	0.75 ± 0.00	0.78 ± 0.00	0.72 ± 0.01	0.0001***
Trochanter BMD (gm/cm2)	0.70 ± 0.00	0.73 ± 0.01	0.66 ± 0.01	0.0001***
Intertrochanter BMD (gm/cm2)	1.10 ± 0.01	1.16 ± 0.01	1.04 ± 0.01	0.0001***
Total spine BMD (gm/cm2)	0.99 ± 0.01	1.05 ± 0.01	0.96 ± 0.01	0.0002***
L1 BMD (gm/cm2)	0.96 ± 0.01	1.00 ± 0.01	0.92 ± 0.01	<0.0001***
L2 BMD (gm/cm2)	1.02 ± 0.01	1.06 ± 0.01	0.99 ± 0.01	<0.0001***
L3 BMD (gm/cm2)	1.05 ± 0.01	1.09 ± 0.01	1.01 ± 0.01	0.0001***
L4 BMD (gm/cm2)	1.05 ± 0.01	1.08 ± 0.02	1.02 ± 0.01	0.0002***
High sensitivity CRP	3.97 ± 0.32	4.69 ± 0.39	3.36 ± 0.46	0.0372*
Overweight (%)	74.73+1.9	97.54 ± 0.89	55.51 ± 2.91	<0.0001***
Abdominal obesity (%)	65.52+2.48	89.49 ± 1.6	45.44 ± 3.39	<0.0001***
Hypertriglyceridemia (%)	61.37+2.38	73.61 ± 2.44	51.08 ± 2.95	<0.0001***
Low HDL (%)	56.12+1.97	69.15 ± 2.83	45.18 ± 2.63	<0.0001***
Diabetes (%)	23.66+1.15	37.39 ± 2.62	12.13 ± 1.13	<0.0001***
Prediabetes (%)	22.31+1.46	29.92 ± 2.84	15.92 ± 1.41	0.0005***
Insulin resistance (%)	21.06+1.73	32.07 ± 2.81	11.82 ± 1.2	<0.0001***
Hypertension (%)	68.48+2.13	77.33 ± 3.26	61.05 ± 1.88	0.0006***
COPD (%)	7.38+1	7.88 ± 1.36	6.97 ± 1.35	0.6287
Cancer (%)	18.65+1.41	20.71 ± 1.82	16.91 ± 1.63	0.0617
Use of prednisone or cortisone (%)	8.48+0.83	10.75 ± 1.5	6.58 ± 1.09	0.0535
Family history of osteoporosis (%)	18.36+1.4	17.23 ± 2.12	19.33 ± 1.86	0.4693
Did mother ever fracture hip (%)	8.86+0.94	9.98 ± 1.26	7.91 ± 1.35	0.2854
Did father ever fracture hip (%)	4.79+1.07	4.57 ± 1.4	4.99 ± 1.24	0.7895

Student’s t tests were used for detecting differences in mean values of continuous variables between males and females. Chi-squared tests were used for detecting associations between categorical variables and gender. *P value < 0.05, **P value < 0.01, ***P value < 0.001.

### The Association Between Metabolic Dysfunction-Associated Fatty Liver Disease With Femoral Bone Mineral Density

We performed a multiple linear regression analysis to examine the association between MAFLD and femoral BMD. The regression coefficients (β) and 95% confidence intervals (CIs) were estimated in three models. Higher femoral BMD, including the BMD of the femur, femoral neck, intertrochanter, and trochanter, were found in the patients with MAFLD, even after fully adjusting for confounders (**Table 2**). Nonetheless, when BMI was adjusted in the multiple liner regression analyses, the association between MAFLD femoral BMD also became insignificant. To further examine the association between MAFLD and femoral osteoporosis, we carried out the multiple logistic regression analysis to explore the association between MAFLD related variables and femoral osteoporosis. In the adjusted II model, apart from diabetes, prediabetes, hypertension, hypertriglyceridemia and insulin resistance, all association was observed between the MAFLD related variables and femoral osteoporosis (**Table 3**). As shown in **Table 4**, MAFLD was negatively associated with femoral osteoporosis when BMD was adjusted (OR=0.89, 95% CI: 0.52 to 1.53), although the relationship became insignificant.

**Table 2:** Multiple linear regression models for the association of MAFLD and femoral bone mineral density.

Femoral bone mineral density(gm/cm2)	Models	MAFLD	
		β (95% CI)	P value
**Total femur BMD**	Crude	0.10 (0.08, 0.11)	< 0.0001
	Adjust I	0.08 (0.07, 0.09)	< 0.0001
	Adjust II	0.06 (0.05, 0.08)	< 0.0001
**Femur neck BMD**	Crude	0.06 (0.05, 0.07)	< 0.0001
	Adjust I	0.05 (0.04, 0.06)	< 0.0001
	Adjust II	0.04 (0.03, 0.05)	< 0.0001
**Trochanter BMD**	Crude	0.07 (0.06, 0.09)	< 0.0001
	Adjust I	0.06 (0.05, 0.07)	< 0.0001
	Adjust II	0.05 (0.04, 0.06)	< 0.0001
**Intertrochanter BMD**	Crude	0.11 (0.10, 0.13)	< 0.0001
	Adjust I	0.09 (0.08, 0.11)	< 0.0001
	Adjust II	0.07 (0.06, 0.09)	< 0.0001

Survey-weight adjusted multiple linear regression model were used in this analysis. The adjust I was adjusted for age, sex, race/ethnicity, education, marital status. The adjust II was adjusted for dring habit, uric acid, Phosphorus, alkaline phosphatase, creatinine, alanine aminotransferase, copd, cancer, maternal fracture history, family history of osteoporosis, in addition to adjust I.

**Table 3:** Multiple logistic regression models for the association of MAFLD related variables and osteoporosis.

MAFLD related variables	N	Non-adjusted	Adjust I	Adjust II	
**Overweight**	1499	0.23 (0.16, 0.33)	0.22 (0.15, 0.33)	0.28 (0.18, 0.44)	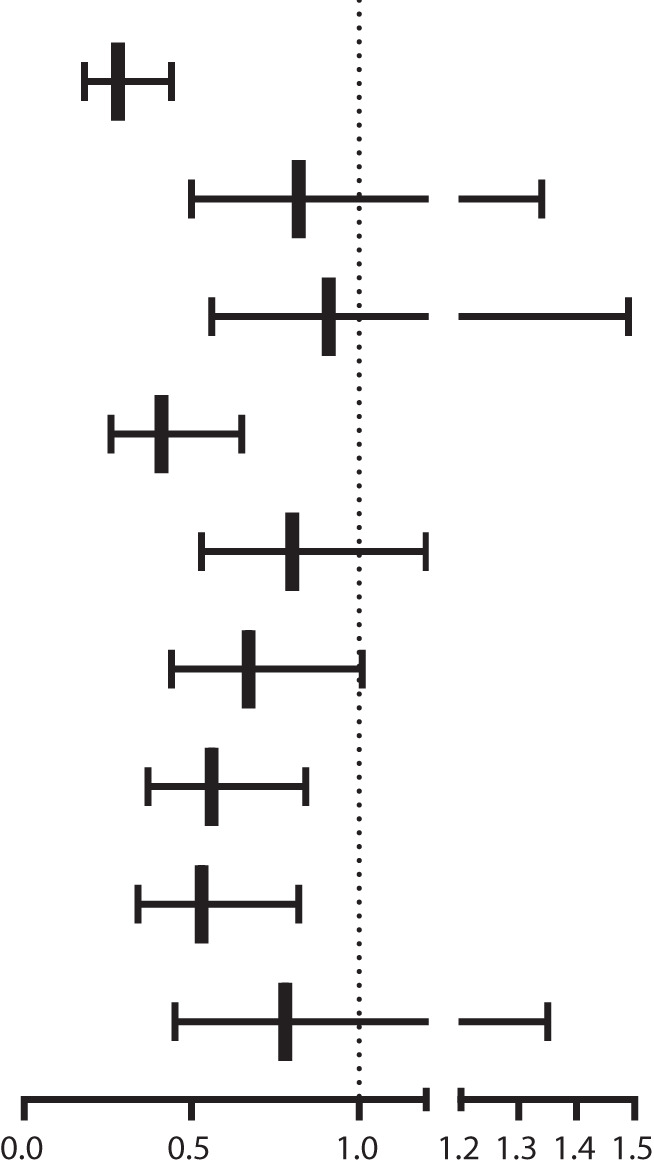
**Diabetes**	605	0.59 (0.39, 0.90)	0.65 (0.42, 1.01)	0.82 (0.50, 1.34)	
**Prediabetes**	480	0.87 (0.58, 1.31)	0.71 (0.46, 1.11)	0.91 (0.56, 1.49)	
**Abdominal obesity**	1250	0.53 (0.38, 0.75)	0.27 (0.18, 0.40)	0.41 (0.26, 0.65)	
**Hypertension**	1061	0.73 (0.52, 1.02)	0.59 (0.41, 0.86)	0.80 (0.53, 1.20)	
**Hypertriglyceridemia**	1258	0.72 (0.51, 1.01)	0.55 (0.38, 0.80)	0.67 (0.44, 1.01)	
**Low HDL**	1212	0.66 (0.47, 0.93)	0.49 (0.34, 0.70)	0.56 (0.37, 0.84)	
**Hyper-hsCRP**	917	0.62 (0.43, 0.88)	0.60 (0.41, 0.88)	0.53 (0.34, 0.82)	
**Insulin resistance**	479	0.56 (0.35, 0.89)	0.64 (0.39, 1.05)	0.78 (0.45, 1.35)	

Survey-weight adjusted multiple logistic regression model were used in this analysis. The adjust I was adjusted for age, sex,race/ethnicity, education, marital status. The adjust II was adjusted for dring habit, uric acid, Phosphorus, alkaline phosphatase,creatinine, alanine aminotransferase, copd, cancer, family history of osteoporosis, in addition to adjust I.

**Table 4:** Subgroup analysis of fully adjusted odd ratios (95% confidence interval) of MAFLD and osteoporosis stratified by sex, age, abdominal obesity, hyper-hscrp and low HDL.

	N	Crude	Adjust I	Adjust II	Adjust II
**Total**	2031	0.33 (0.22, 0.49)	0.37 (0.24, 0.57)	0.49 (0.30, 0.78)	0.89 (0.52, 1.53)
**Gender**					
Man	1070	0.21 (0.08, 0.55)	0.20 (0.07, 0.53)	0.30 (0.10, 0.87)	0.43 (0.12, 1.51)
Female	961	0.39 (0.25, 0.62)	0.44 (0.28, 0.71)	0.57 (0.33, 0.98)	1.07 (0.58, 1.98)
**Age**					
<60	660	0.12 (0.03, 0.51)	0.17 (0.04, 0.76)	0.18 (0.04, 0.94)	0.41 (0.06, 2.99)
≥60, <70	797	0.32 (0.16, 0.66)	0.28 (0.13, 0.59)	0.28 (0.11, 0.69)	0.44 (0.16, 1.17)
≥70	574	0.43 (0.25, 0.74)	0.52 (0.29, 0.92)	0.66 (0.33, 1.29)	1.12 (0.52, 2.42)
**Abdominal obesity**					
No	871	0.27 (0.11, 0.69)	0.55 (0.20, 1.49)	0.88 (0.30, 2.58)	1.29 (0.42, 4.03)
Yes	1250	0.40 (0.24, 0.66)	0.59 (0.35, 1.02)	0.66 (0.36, 1.23)	0.79 (0.41, 1.51)
**Hyper-hscrp**					
No	1114	0.34 (0.20, 0.60)	0.42 (0.23, 0.75)	0.60 (0.30, 1.17)	0.99 (0.47, 2.11)
Yes	917	0.35 (0.19, 0.64)	0.37 (0.20, 0.70)	0.47 (0.23, 0.95)	0.83 (0.37, 1.87)
**Low HDL**					
No	819	0.28 (0.14, 0.56)	0.30 (0.14, 0.61)	0.44 (0.20, 0.98)	0.69 (0.28, 1.72)
Yes	1212	0.38 (0.23, 0.63)	0.48 (0.28, 0.83)	0.60 (0.32, 1.11)	1.12 (0.55, 2.27)

Survey-weight adjusted multiple linear regression model were used in this analysis. The adjust I was adjusted for age, sex, race/ethnicity, education, marital status. The adjust II was adjusted for dring habit, uric acid, Phosphorus, alkaline phosphatase, creatinine, alanine aminotransferase, copd, cancer, family history of osteoporosis, in addition to adjust I. The adjust III was adjusted for body mass index, in addition to adjust II.

### Subgroup Analyses of Factors Influencing the Association Between MAFLD and Femoral Osteoporosis

Subsequently, subgroup analysis was performed to further understand the association between MAFLD and femoral osteoporosis (**Table 4**). In subgroup analyses stratified by sex, a significantly negative association between MAFLD and femoral osteoporosis was observed in both men and women. When stratified by age (<60, 60 to 70, and ≥70 years), abdominal obesity (yes or no), hyper-hs CRP (yes or no) and Low HDL (yes or no), we could still observe a negative association between MAFLD and femoral osteoporosis, although the correlation becomes tenuous. Furthermore, in subgroup analyses stratified by age, we found that the protective effect of the higher age group on osteoporosis was decreased compared with those younger than 60 years, which is consistent with a previous research suggesting that aging is a risk factor for osteoporosis. Nonetheless, when BMI was adjusted in the subgroup analyses, the association between MAFLD and femoral osteoporosis became insignificant.

### The Association Between Liver Stiffness and Femoral Bone Mineral Density

We explored the relationship between different grades of liver stiffness and femoral BMD in the multiple linear regression. We found that in the crude, adjust I and adjust II model, patients with significant liver stiffness (fibrosis grade ≥ F2) had higher femoral BMD levels. Besides, this result was still observed in patients with severe liver stiffness (fibrosis grade ≥ F3) or cirrhosis (fibrosis grade ≥ F4). Nonetheless, when BMI was further adjusted in the adjust III model, the relationship between significant liver stiffness, severe liver stiffness, cirrhosis and femoral osteoporosis became insignificant (showed in the **Supplementary Table 1**).

### Sensitivity Analysis

In the sensitivity analysis, we did the following things. First, we explored the relationship between MAFLD and whole osteoporosis (include femoral and lumbar osteoporosis). As showed in the **Supplementary Table 2**, those patients with MAFLD had a higher lumbar BMD,when adjusted the confounders but not included BMI, compare with their counterparts. Nonetheless, the lumbar BMD of patients with MAFLD was parallel with those without MAFLD, when adjusted BMI. Besides, similar to the relationship between MAFLD and femoral osteoporosis, the relationship between MAFLD and whole osteoporosis was showed in adjust II model that patients with MAFLD had a lower risk ratio for whole osteoporosis compared to those without MAFLD (OR=0.46, P value=0.0022), and then once BMI was adjusted, the relationship became insignificant (OR=0.81, P value=0.4539). (showed in the **Supplementary Table 3**).

Second, the relationship between liver stiffness and whole osteoporosis was showed in the **Supplementary Table 4**. There was no association between liver fibrosis and whole osteoporosis, regardless of adjusting BMI. These results may suggest that patients with MAFLD and liver fibrosis have a higher BMD and a lower risk ratio for osteoporosis, but may be confounded by BMI.

## Discussion

In this non-institutionalized general population study among the United States over 50 years old, we explored the association of MAFLD and BMD. The results showed that the prevalence of MAFLD between men and women was accounting for 50.3% and 39.6%, respectively. MAFLD was associated with a significantly higher femoral BMD, including total femur BMD, femur neck BMD, trochanter BMD and intertrochanter BMD, which might reduce the risk of osteoporosis, after adjusting for a serious of underlying confounders. However, when BMI was additional included in the liner regression models (adjust III model), the association between MAFLD and femoral BMD became so weak that the association between MAFLD and osteoporosis became insignificant.

The association between NAFLD and osteoporosis had been extensively studied in many cross-sectional and prospective cohort studies (32–35). Nevertheless, the link between NAFLD and BMD is still controversial. Stefano Ciardullo et al. (32) performed a cross-sectional analysis of the association between NAFLD and liver fibrosis with osteoporosis based on the data from NHANES 2017-2018. They found neither NAFLD nor fibrosis was not association with osteoporosis in US population age over than 50 years. Xie et al. also used NHANES 2017-2018 data but in US population aged 20 to 59 years (33). They found a negative correlation between NAFLD and lumbar bone mineral density. But when adjust BMI, the association between NAFLD and osteoporosis also became insignificant, even stratified by sex, age and race. Although recent studies have shown that patients with MAFLD tend to have a worse prognosis than those with NAFLD, our conclusion was aligned with the result of Stefano Ciardullo et al., which may mean that either NAFLD or MAFLD may exert a similar and weak influence on femoral bone mineral density (may mainly due to BMI). However, the study conducted by Xie et al. was on people aged 20-59 years and investigated the lumbar BMD, which may cause the different result of our study.

In this study, the BMI was significantly high in MAFLD patients relative to those without MAFLD. The beneficial effect of MAFLD on bone mineral density may be due to the higher BMI of the subjects leading to greater bone load in the patients, which stimulates bone mass accumulation (36).

Collectively, BMI is a worthy confounding factor to consider, without considering it, the study outcomes or arrive at erroneous conclusions. In addition, studies of Higher BMD in patients with metabolic syndrome (MetS) were reported in several studies (37, 38), but the association between MetS and higher BMD will disappear or reverse when BMI is adjusted. Therefore, in this study, we suggest that the protective effect of MAFLD on bone mineral density is at least partially driven by the higher mechanical load in patients with MAFLD.

Although little is known about the underlying mechanisms of chronic liver disease and osteoporosis, there is some evidence that NAFLD may increase the risk of osteoporosis and that more severe histological conditions are associated with lower BMD (11, 39–41). The pathophysiological interrelationships between NAFLD and low BMD may involve chronic low-grade inflammation and markers of bone metabolism. On the one hand, greater tumor necrosis factor (TNF)-α levels were observed in NAFLD patients, which were involved in both stimulation of osteoclast formation and inhibition of osteoblast activation by their progenitor cells (42). On the other hand, Osteopontin (OPN), Osteoprotegerin (OPG), Osteocalcin and other markers of bone metabolism have been (43) proved to play an important role in chronic liver disease and osteoporosis (44–47). It cannot be ruled out that patients with more severe steatosis have a higher BMI, which may reverse the harmful effects of steatosis on osteoporosis.

However, MAFLD involves many facets, not only T2DM and obesity, and could be termed a multisystem disease. The links between MAFLD and the risk of osteoporosis might be complicated and require further investigation to unveil the potential mechanisms.

MAFLD is a constellation of interrelated clinical, metabolic, physiological and biochemical factors. Due to the clinical features of obesity and abdominal obesity, it may have higher levels of inflammatory cytokines and adipokines (eg, interleukin-1, interleukin-6, C-reactive protein, tumor necrosis factor alpha) (48). Studies have shown that these inflammatory cytokines and adipokines are involved in bone resorption, which may increase the risk of osteoporosis (48, 49). Therefore, it remains unclear whether higher BMI and T2DM morbidity can translate into a protective factor for fracture. Although our and others studies have found that obesity and T2DM protect against osteoporosis (50), studies have shown that adults with obesity or T2DM are at higher risk of falls and fractures compared with normal-weight or non-T2DM patients (51). Hence, the impact of MAFLD-related diseases on fracture risk may inconsistent. For example, pre-diabetes was considered to be a protective factor for fracture (52, 53), while Mets, T2DM, insulin resistance, higher body mass index are considered to increase fracture risk (53–56). This might explain this heterogeneous complex relationship. However, more studies need to be conducted in different populations and countries to fully elucidate the pathophysiology behind this complexity.

Several limitations should, however, be considered. First, this cross-sectional design failed to establish temporal relationships or causality between the MAFLD and osteoporosis. Second, we used VCTE to diagnose fatty liver in the study. But although liver biopsy is the gold standard for diagnosing fatty liver, several evidences (29, 57) had validated the diagnostic accuracy of VCTE as a favorable proxy indicator in identifying any degree of hepatic steatosis. Besides, our study was conducted in the United States among people aged over 50, so the results may not be applied in other countries. In addition, as our data lacked the results of an oral glucose-tolerance test, it might reduce the prevalence assessment of T2DM and would attenuate our findings. It is important to note that our goal was to evaluate the association of MAFLD with osteoporosis and not with fracture per se. However, fracture is a main endpoint of osteoporosis. The results displayed herein hence are not generalized to fracture study.

In conclusion, in this cross-sectional study of people over 50 years old in the United States, we found that patients with MAFLD had higher BMD in despite of the fact that this may be contributed by higher BMI. However, this does not mean that these patients have a lower fracture risk. Therefore, prospective studies of MAFLD and fracture are required to elucidate the causal relationship between MAFLD and fracture.

## Data Availability Statement

The original contributions presented in the study are included in the article/**Supplementary Material**, further inquiries can be directed to the corresponding author.

## Ethics Statement

Ethical review and approval was not required for the study on human participants in accordance with the local legislation and institutional requirements. The patients/participants provided their written informed consent to participate in this study. Written informed consent was obtained from the individual(s) for the publication of any potentially identifiable images or data included in this article.

## Author Contributions

RL, YZ, HJL, HCL, and LL made substantial contributions to the conception and design of the study. HJL, HCL, and LL analyzed the data. HJL and HCL drafted the manuscript. HJL and HCL revised the manuscript. All the authors assisted in the acquisition and interpretation of data, contributed to the critical revision of the manuscript for important intellectual content, and approved the final version.

## Funding

This work was supported by the Natural Science Foundation of Guangdong Province (N0. 2021A1515111148) and Medical Science and Technology Research Foundation of Guangdong Province (N0. 20211117145113732).

## Conflict of Interest

The authors declare that the research was conducted in the absence of any commercial or financial relationships that could be construed as a potential conflict of interest.

## Publisher’s Note

All claims expressed in this article are solely those of the authors and do not necessarily represent those of their affiliated organizations, or those of the publisher, the editors and the reviewers. Any product that may be evaluated in this article, or claim that may be made by its manufacturer, is not guaranteed or endorsed by the publisher.
