# Supplementary material for: Association of Metabolic Dysfunction-Associated Fatty Liver Disease and Liver Stiffness With Bone Mineral Density in American Adults
Source: Front Endocrinol (Lausanne). 2022 Jun 30;13:891382. doi: 10.3389/fendo.2022.891382 (PMC9280639; doi:10.3389/fendo.2022.891382)
Supplement: Supplementary file 1 [file DataSheet_1.zip › Supplementary Table 1.docx]

**Supplementary table 1.** Multiple linear regression models for the association of liver fibrosis and bone mineral density.

| **Bone mineral density**  **(gm/cm2)** | **Significant liver fibrosis**  **(fibrosis grade ≥ F2)** | | **Severe liver fibrosis**  **(fibrosis grade ≥ F3)** | | **Cirrhosis**  **(fibrosis grade ≥ F4)** | | |
| --- | --- | --- | --- | --- | --- | --- | --- |
|  | **Models** | **β** (95% CI) | **Models** | **β** (95% CI) | **Models** | **β** (95% CI) |  |
| **Total femur BMD** | Crude | 0.06 (0.03, 0.08) | Crude | 0.06 (0.04, 0.09) | Crude | 0.04 (0.01, 0.08) |  |
|  | Adjust I | 0.04 (0.02, 0.06) | Adjust I | 0.05 (0.03, 0.07) | Adjust I | 0.04 (0.01, 0.07) |  |
|  | Adjust II | 0.06 (0.04, 0.09) | Adjust II | 0.05 (0.03, 0.08) | Adjust II | 0.05 (0.02, 0.09) |  |
|  | Adjust III | 0.04 (0.01, 0.08) | Adjust III | 0.02 (-0.01, 0.04) | Adjust III | 0.03 (-0.00, 0.06) |  |
| **Femur neck BMD** | Crude | 0.03 (0.01, 0.05) | Crude | 0.03 (0.01, 0.06) | Crude | 0.03 (-0.01, 0.06) |  |
|  | Adjust I | 0.02 (0.00, 0.04) | Adjust I | 0.03 (0.01, 0.05) | Adjust I | 0.03 (-0.00, 0.06) |  |
|  | Adjust II | 0.02 (0.00, 0.04) | Adjust II | 0.03 (0.01, 0.05) | Adjust II | 0.04 (0.01, 0.08) |  |
|  | Adjust III | -0.01 (-0.02, 0.01) | Adjust III | 0.00 (-0.02, 0.02) | Adjust III | 0.02 (-0.01, 0.05) |  |
| **Trochanter BMD** | Crude | 0.05 (0.03, 0.07) | Crude | 0.06 (0.04, 0.08) | Crude | 0.05 (0.02, 0.08) |  |
|  | Adjust I | 0.04 (0.02, 0.06) | Adjust I | 0.05 (0.04, 0.07) | Adjust I | 0.05 (0.02, 0.08) |  |
|  | Adjust II | 0.04 (0.02, 0.05) | Adjust II | 0.06 (0.04, 0.08) | Adjust II | 0.06 (0.03, 0.09) |  |
|  | Adjust III | 0.01 (-0.00, 0.03) | Adjust III | 0.03 (0.01, 0.05) | Adjust III | 0.04 (0.01, 0.07) |  |
| **Intertrochanter BMD** | Crude | 0.06 (0.03, 0.08) | Crude | 0.06 (0.03, 0.09) | Crude | 0.04 (-0.01, 0.09) |  |
|  | Adjust I | 0.04 (0.01, 0.06) | Adjust I | 0.05 (0.02, 0.07) | Adjust I | 0.03 (-0.01, 0.07) |  |
|  | Adjust II | 0.04 (0.01, 0.06) | Adjust II | 0.05 (0.02, 0.08) | Adjust II | 0.05 (0.01, 0.10) |  |
|  | Adjust III | -0.00 (-0.03, 0.02) | Adjust III | 0.01 (-0.02, 0.03) | Adjust III | 0.02 (-0.02, 0.06) |  |
| **Total spine BMD** | Crude | 0.09 (0.05, 0.12) | Crude | 0.07 (0.03, 0.11) | Crude | 0.03 (-0.03, 0.09) |  |
|  | Adjust I | 0.06 (0.03, 0.09) | Adjust I | 0.05 (0.02, 0.09) | Adjust I | 0.03 (-0.02, 0.09) |  |
|  | Adjust II | 0.06 (0.03, 0.10) | Adjust II | 0.06 (0.02, 0.10) | Adjust II | 0.06 (0.01, 0.12) |  |
|  | Adjust III | 0.02 (-0.01, 0.06) | Adjust III | 0.02 (-0.02, 0.05 | Adjust III | 0.04 (-0.01, 0.10) |  |
| **L1 BMD** | Crude | 0.08 (0.06, 0.11) | Crude | 0.10 (0.06, 0.13) | Crude | 0.08 (0.03, 0.12) |  |
|  | Adjust I | 0.07 (0.04, 0.09) | Adjust I | 0.08 (0.05, 0.11) | Adjust I | 0.08 (0.03, 0.12) |  |
|  | Adjust II | 0.07 (0.04, 0.09) | Adjust II | 0.08 (0.05, 0.11) | Adjust II | 0.09 (0.05, 0.14 |  |
|  | Adjust III | 0.04 (0.01, 0.06) | Adjust III | 0.04 (0.01, 0.07) | Adjust III | 0.07 (0.02, 0.11) |  |
| **L2 BMD** | Crude | 0.07 (0.04, 0.10) | Crude | 0.08 (0.05, 0.11) | Crude | 0.04 (-0.01, 0.09) |  |
|  | Adjust I | 0.05 (0.02, 0.07) | Adjust I | 0.06 (0.03, 0.09) | Adjust I | 0.05 (-0.00, 0.09) |  |
|  | Adjust II | 0.05 (0.02, 0.08) | Adjust II | 0.07 (0.03, 0.10) | Adjust II | 0.07 (0.02, 0.12) |  |
|  | Adjust III | 0.02 (-0.01, 0.05) | Adjust III | 0.03 (-0.00, 0.07) | Adjust III | 0.05 (-0.00, 0.10) |  |
| **L3 BMD** | Crude | 0.06 (0.03, 0.09) | Crude | 0.07 (0.03, 0.10) | Crude | 0.03 (-0.02, 0.08) |  |
|  | Adjust I | 0.05 (0.02, 0.07) | Adjust I | 0.06 (0.02, 0.09) | Adjust I | 0.04 (-0.01, 0.09) |  |
|  | Adjust II | 0.05 (0.02, 0.08) | Adjust II | 0.06 (0.02, 0.09) | Adjust II | 0.06 (0.01, 0.11) |  |
|  | Adjust III | 0.01 (-0.02, 0.04) | Adjust III | 0.02 (-0.01, 0.06) | Adjust III | 0.04 (-0.01, 0.09) |  |
| **L4 BMD** | Crude | 0.07 (0.04, 0.11) | Crude | 0.07 (0.03, 0.10) | Crude | 0.01 (-0.05, 0.06) |  |
|  | Adjust I | 0.05 (0.02, 0.08) | Adjust I | 0.05 (0.02, 0.09) | Adjust I | 0.01 (-0.04, 0.06) |  |
|  | Adjust II | 0.06 (0.03, 0.09) | Adjust II | 0.06 (0.02, 0.09) | Adjust II | 0.05 (-0.01, 0.11) |  |
|  | Adjust III | 0.02 (-0.01, 0.05) | Adjust III | 0.02 (-0.02, 0.05) | Adjust III | 0.02 (-0.03, 0.08) |  |
