# Supplementary material for: Association of Metabolic Dysfunction-Associated Fatty Liver Disease and Liver Stiffness With Bone Mineral Density in American Adults
Source: Front Endocrinol (Lausanne). 2022 Jun 30;13:891382. doi: 10.3389/fendo.2022.891382 (PMC9280639; doi:10.3389/fendo.2022.891382)
Supplement: Supplementary file 1 [file DataSheet_1.zip › Supplementary Table 4.docx]

**Supplementary table 4.** Multiple linear regression models for the association of liver fibrosis and osteoporosis (femoral and lumbar).

| **Exposure** | **Models** | | | | |
| --- | --- | --- | --- | --- | --- |
|  | **N** | **Crude**  **OR (95% CI)** | **Adjust I**  **OR (95% CI)** | **Adjust II**  **OR (95% CI)** | **Adjust III**  **OR (95% CI)** |
| **Significant liver fibrosis**  **(fibrosis grade ≥ F2)** |  |  |  |  |  |
| LSM < 8.2 KPa | 1801 | Reference | Reference | Reference | Reference |
| LSM ≥ 8.2 KPa | 230 | 0.61 (0.36, 1.06) | 0.72 (0.40, 1.28) | 0.67 (0.33, 1.37) | 0.86 (0.41, 1.82) |
| **Severe liver fibrosis**  **(fibrosis grade ≥ F3)** |  |  |  |  |  |
| LSM < 9.7 KPa | 1878 | Reference | Reference | Reference | Reference |
| LSM ≥ 9.7 KPa | 153 | 0.56 (0.28, 1.11) | 0.62 (0.30, 1.28) | 0.61 (0.26, 1.47) | 0.85 (0.34, 2.11) |
| **Cirrhosis**  **(fibrosis grade ≥ F4)** |  |  |  |  |  |
| LSM < 13.6 KPa | 1965 | Reference | Reference | Reference | Reference |
| LSM ≥ 13.6 KPa | 66 | 0.75 (0.30, 1.88) | 0.79 (0.30, 2.07) | 0.74 (0.23, 2.37) | 0.95 (0.28, 3.25) |

**Supplementary table 4.** Multiple linear regression models for the association of liver fibrosis and spine osteoporosis.

| **Exposure** | **Models** | | | | |
| --- | --- | --- | --- | --- | --- |
|  | **N** | **Crude**  **OR (95% CI)** | **Adjust I**  **OR (95% CI)** | **Adjust II**  **OR (95% CI)** | **Adjust III**  **OR (95% CI)** |
| **Significant liver fibrosis**  **(fibrosis grade ≥ F2)** |  |  |  |  |  |
| LSM < 8.2 KPa | 952 | Reference | Reference | Reference | Reference |
| LSM ≥ 8.2 KPa | 112 | 0.44 (0.21, 0.92) | 0.23 (0.07, 0.75) | 0.23 (0.07, 0.75) | 0.33 (0.09, 1.15) |
| **Severe liver fibrosis**  **(fibrosis grade ≥ F3)** |  |  |  |  |  |
| LSM < 9.7 KPa | 988 | Reference | Reference | Reference | Reference |
| LSM ≥ 9.7 KPa | 76 | 0.41 (0.16, 1.03) | 0.21 (0.05, 0.84) | 0.21 (0.05, 0.84) | 0.32 (0.08, 1.38) |
| **Cirrhosis**  **(fibrosis grade ≥ F4)** |  |  |  |  |  |
| LSM < 13.6 KPa | 1032 | Reference | Reference | Reference | Reference |
| LSM ≥ 13.6 KPa | 32 | 0.87 (0.30, 2.51) | 0.43 (0.10, 1.92) | 0.43 (0.10, 1.92) | 0.55 (0.11, 2.67) |

Survey-weight adjusted multiple linear regression model were used in this analysis. The adjust I was adjusted for age, sex, race/ethnicity, education, marital status. The adjust II was adjusted for dring habit, uric acid, Phosphorus, alkaline phosphatase, creatinine, alanine aminotransferase, copd, cancer, family history of osteoporosis, in addition to adjust I. The adjust III was adjusted for body mass index, in addition to adjust II.
